# Characterisation of *Alternaria alternata* Allergoids: Evaluation of the Stability of Grass Pollen Allergen Extracts Mixed with *Alternaria alternata* Allergoids

**DOI:** 10.3390/jof11030181

**Published:** 2025-02-25

**Authors:** Eva Abel-Fernández, Enrique Fernández-Caldas, Salvador Iborra

**Affiliations:** Inmunotek S. L., 28805 Alcalá de Henares, Madrid, Spain; efcaldas@inmunotek.com (E.F.-C.); siborra@inmunotek.com (S.I.)

**Keywords:** *Alternaria alternata*, fungi allergy, allergen specific immunotherapy, allergen extract, allergoid

## Abstract

Allergens from pollen, mites, and moulds often sensitise patients simultaneously, posing challenges for developing stable and effective combination vaccines. *Alternaria alternata*, a major source of indoor and outdoor allergens, is strongly linked to asthma development and contains proteolytic enzymes that can degrade other allergens, potentially reducing vaccine efficacy. This study aimed to evaluate the safety, efficacy, and stability of polymerised *A. alternata* extracts (allergoids) compared to native extracts and their compatibility with pollen extracts (*Phleum pratense*). Allergoids were prepared using glutaraldehyde and characterised through SDS-PAGE, LC-MS/MS, NMR, and gas chromatography. Their immunogenicity and IgE-binding properties were assessed via Western blot and ELISA competition assays, while enzymatic activity was analysed using ApiZym kits. Mice immunisation experiments were conducted to evaluate antibody responses. Polymerised extracts exhibited reduced IgE-binding capacity while maintaining IgG-binding and immunogenicity. Mice immunised with allergoids generated antibodies that efficiently blocked IgE binding in allergic patients. Proteolytic activity was significantly reduced in allergoids, and pollen extracts remained stable when combined with them. These findings demonstrate that *A. alternata* allergoids are a safer, more stable alternative for immunotherapy, offering enhanced efficacy and reduced injections for polysensitised patients. This study provides critical insights for designing optimised combination vaccines.

## 1. Introduction

Fungi are the fourth most frequent cause of allergic respiratory diseases and are particularly associated with poor asthma prognosis [1,2]. Allergic immunotherapy (AIT) reduces the risk of developing asthma in children with allergenic rhinitis due to pollen and mites [3,4,5]. However, despite the long-standing use of AIT with mould extracts, evidence supporting its efficacy in treating respiratory allergic diseases remains limited [6,7]. Furthermore, immunotherapy for mould allergies is not completely exempt from risks. The administration of specific native fungal protein extracts carries a high risk of adverse reactions [8,9,10]. Consequently, several allergy and asthma organisations did not recommend AIT for fungi in children due to insufficient evidence regarding the efficacy and safety of native fungal extracts [11,12].

One of the primary goals of AIT is to increase safety by minimising secondary adverse events while preserving efficacy [13,14]. The production of allergoids, or polymerised allergenic extracts with glutaraldehyde, supposes a valid alternative to AIT with unmodified extracts. Due to their reduced allergenicity, while retaining immunogenicity, polymerised extracts (PEs) are widely used in the treatment of pollen and mite allergies, offering an efficient and safer alternative to conventional native unmodified extracts. The chemical cross-linking of allergens during polymerisation produces larger molecules, which reduces accessibility to or the recognition of IgE epitopes (decreasing allergenicity) while preserving IgG and linear T-cell epitopes (ensuring immunogenicity). This property of allergoids allows for faster up-dosing during immunotherapy without increasing the risk of adverse reactions [15]. Numerous allergens presenting proteolytic activity in different allergenic sources, mainly fungi, mites and cockroaches, which are particularly rich in proteases have been described [16]. Over 50% of the allergens with proteolytic activity accepted by the WHO/IUIS are derived from moulds, with serine proteases being the most prominent group [17]. The allergenicity of these enzymatic proteins is thought to be linked to the development of IgE-mediated immune responses (Th2), possibly as an evolved defence mechanism against parasitic infections that depend on enzymes for tissue invasion and migration [18,19]. Mould allergens, in particular, can exacerbate asthma due to their enzymatic activity. This activity disrupts protein junctions within epithelial cells, facilitating the entry of other allergens across epithelial barriers [20,21,22,23,24,25,26]. Furthermore, the enzymatic properties of these proteins can alter the allergenicity and immunogenicity of other proteins by modifying their structure and carbohydrate content [9].

PEs have demonstrated their safety and efficacy in various studies involving pollens, mites, and animal epithelium allergens, benefiting both adult and paediatric patients [27,28,29,30,31,32,33,34]. The European Survey on Adverse Systemic Reactions in Allergen Immunotherapy (EASSI) reported a lower incidence and risk of systemic reactions during subcutaneous immunotherapy (SCIT) with allergoids compared to native extracts [35]. While allergoids have been developed for several allergen sources, their application to fungal allergens has been limited until recently [36,37].

Thus, allergic patients can benefit from allergen-specific immunotherapy using allergoids. Nevertheless, most patients are sensitised to two or more allergenic sources, making it necessary to receive immunotherapy for multiple allergens. For some years now, there has been a debate regarding the use of allergen mixtures (the American approach) versus single or few allergens (the European approach). In the U.S., allergy organisations support the practice of mixing multiple allergens to address polysensitised patients despite concerns about potential allergen degradation and reduced efficacy [38]. In contrast, the European guidelines recommend the use of single allergens or well-characterised mixtures, prioritising standardisation and clinical evidence (EAACI, 2018). These differing perspectives are particularly relevant when evaluating the stability of allergen mixtures in AIT.

The aim of this study was to develop and characterise polymerised allergenic extracts from the mould *Alternaria alternata*, conducting initial trials to assess their safety and efficacy potential. Additionally, we assessed the stability of grass pollen (*Phleum pratense*) in a mould–grass allergoid mixture compared to its combination with native unmodified *A. alternata* extracts. These findings provide valuable insights into the feasibility of integrating mould allergen extracts into AIT formulations alongside other allergen sources without compromising their immunogenic properties.

## 2. Materials and Methods

### 2.1. A. alternata Allergen Extraction and Polymerisation

Metabolic and somatic phases from *A. alternata* cultures (Inmunotek Laboratories, Madrid, Spain) were obtained and extracted in 1/20 *w*/*v* 0.01 M PBS under magnetic continuous stirring for 40 h at 4 °C. The resulting supernatant was filtered through 0.2 µm membranes (Sartorius Stedim Biotech S. A., Aubagne, France) and diafiltrated by tangential ultrafiltration using a 5 kDa polyether sulfone Omega membrane (Pall Life Sciences, Portsmouth, UK) to remove low-molecular-weight impurities. The resulting purified extracts were freeze-dried (native extracts, NE) and subsequently reconstituted in PBS and polymerised with glutaraldehyde 25% (Sigma-Aldrich, St. Louis, MO, USA). The reactions were performed during 18 h at 4 °C in continuous stirring and stopped by the addition of 1.25 M glycine. The resulting allergoids (polymerised extract, PE) were diafiltered through a 100 kDa membrane to remove non-polymerised molecules. The allergoids were lyophilised and kept at 4 °C until use. The protein content of the native extracts and allergoids was measured by the Bradford protein assay (Bio-Rad Laboratories, Madrid, Spain).

### 2.2. Analysis of the Protein and Allergenic Profile of the Extracts: SDS-PAGE and Immunoblotting

The native and polymerised extracts (NE and PE, respectively) were separated by SDS-PAGE under reducing conditions using a Mini-PROTEAN II device (Bio-Rad). Ten micrograms of the sample was loaded per lane and separated in a custom polyacrylamide gel (Any kD Mini-PROTEAN TGX Stain-Free, Bio-Rad) at 300 volts. The gels were stained with GelCode Blue Stain Reagent (ThermoFisher Scientific, Madrid, Spain). Precision Plus Protein All Blue Standards (Bio-Rad) was used as a molecular weight marker. For immunoblotting, proteins were electrotransferred to nitrocellulose membranes using a Trans-Blot Semi-Dry Electrophoresis Transfer Cell at 20 v for 30 min (BioRad). After blocking with 5% bovine serum albumin in PBS-0.25% Tween 20 (PBS-T), the membranes were incubated with a commercial 1:4 diluted serum pool prepared with the sera from patients sensitised to *Alternaria alternata* (Plasmalab International, Everett, WA, USA) for 16 h at 4 °C. In IgE inhibition Western blot assays, this incubation step with sera from sensitised patients was preceded by incubation with 1:10,000 diluted sera from mice immunised for 4 h. Afterwards, the membranes were incubated with 1:2000 diluted horseradish peroxidase (HRP)-conjugated mouse anti-human IgE Fc (Southern Biotech, Birmingham, AL, USA) for 1 h at 4 °C. After washing with PBS-T, the membranes were visualised using Amersham ECL Prime Western blotting Detection Reagent (Amersham, GE Healthcare, Little Chalfont, UK) in a GeneGnome device (Syngene, Cambridge, UK). Precision Plus Protein WesternC Standards (Bio-Rad) in the presence of Precision Protein StrepTactinHRP Conjugate (Bio-Rad) was used as molecular weight markers.

### 2.3. Physicochemical Analysis and Chemical Composition of the Extracts: Analysis of Amino Acid Content, Nuclear Magnetic Resonance, and Mass Spectrometry

The amino acid composition of the native and polymerised extracts was analysed by Gas chromatography and compared to evaluate the presence of free amino acids in the NE and their implication in the polymerisation process. NE and PE were first hydrolysed with 6N HCl under vacuum at 110 °C for 24 h. Afterwards, the amino acid content was assessed in a quantitative amino acid analyser (Biochrom 30; Biochrom Ltd., Cambridge, UK). Norleucine was used as an internal standard.

Nuclear Magnetic Resonance (NMR) was conducted to evaluate the molecular size of the NE and PE. The spectra were obtained for the native extracts and allergoid samples in D_2_O at 298 K in a Bruker Avance 600 MHz spectrometer (Bruker Ltd., Karlsruhe, Germany). The two-dimension diffusion-ordered spectroscopy (2D-DOSY) experiments were developed by recording 64–128 scans for each gradient step, a linear gradient of 16 steps between 2% and 95%, a diffusion time (D) between 0.2 and 0.4 s, and length of the diffusion encoding gradient pulses (δ) between 2 and 4 ms. The spectra were processed with the protocols implemented in the TopSpin 3.0 software (Bruker Ltd.).

The protein content in the native and polymerised extracts was determined by liquid chromatography–mass spectrometry (LC-MS/MS). Briefly, the samples were first precipitated with methanol/chloroform and submitted to a reduction–alkylation process (50 mM TCEP and 200 mM MMTS) before a digestion step with trypsin (1:20 enzyme/protein). The digested samples were then purified in a SEP-PAK C18 column and peptides were separated, depending on their polarity, by liquid chromatography (90 min, reverse phase column C-18). The eluted peptides obtained were fragmented in a mass spectrometer (5600 TRIPLE-TOF, Sciex, Framingham, MA, USA). An NCBI database search was performed with the data obtained through the MASCOT search engine. The sequences of the proteins identified with at least two peptides and presenting a protein score superior to 20 were compared with the WHO/IUIS protein database.

### 2.4. Analysis of the Allergenic Activity: IgE/IgG ELISA Competition Assays

The biological IgE and IgG potency of the *A. alternata* native extracts and their corresponding allergoids (50% inhibition) were determined by ELISA competition assays. Microplates were coated o/n with 1 μg of the native extract (NE) or polymerised extract (PE) in 0.05 M carbonate/bicarbonate buffer (pH 9.6). In total, 50 μL of serially diluted competitors and 50 μL of human sera pool from patients sensitised to *A. alternata* (Plasmalab International) were added to the wells and incubated o/n at 4 °C (1:4 diluted for IgE and 1:1024 for IgG binding detection). After washing, the microplates were incubated with secondary antibody (1:2000 diluted HRP-conjugated mouse anti-human IgE Fc; Southern Biotech, or 1:5000 diluted HRP-conjugated rabbit anti-human IgG Fc) for 1 h. Finally, the microplates were washed and incubated with o-phenylenediamine dihydrochloride (OPD, Sigma-Aldrich) for 30 min. The reaction was stopped with 1/10 HCl and absorbance was read at 492 nm. All the samples were assayed in duplicate. Blank controls were included in the assays and non-inhibition controls (sera pool without competitor) were included. Percent inhibition was calculated using the following equation: percent inhibition = 100 − [(OD of serum with competitor/OD of serum without competitor) × 100].

### 2.5. Analysis of the Immunogenicity: Mice Immunisation

Eighteen BALB/c mice were divided into 3 groups (six mice each) and were immunised: (1) control group: immunised with SPB, (2) NE group: immunised with 20 µg of NE total protein, and (3) PE group: immunised with 20 µg of PE total protein. In total, the animals received four subcutaneous injections every two weeks. Seven days after the last immunisation, blood was collected, and the mice were sacrificed. To determine the specific IgG production induced by immunisation with NE or PE, the total specific IgG levels were analysed in the sera of the three groups of mice. ELISA plates were coated with NE or PE overnight. Afterwards, sera were added and left to stand overnight. For the detection of total IgG, sera were diluted 1:10,000 and incubated o/n. Bound antibodies were detected with 1:2000 diluted HRP-conjugated goat anti-mouse IgG (Bethyl Laboratories, Montgomery, TX, USA). OPD was added and the reaction was measured at 492 nm.

To analyse the IgE blocking capacity of the IgG antibodies, Western blot inhibition assays were developed. The assays consisted of the incubation of membranes with 1:10,000 diluted mice sera (immunised with NE or PE) to allow the union of IgG and consecutively, the incubation with the commercial (human) sera pool, from patients sensitised to *A. alternata*. After washing with SPBT, the membranes were incubated for 2 h with 1:2000 diluted HRP-conjugated mouse anti-human IgE Fc (SouthernBiotech, Birmingham, AL, USA) and developed as previously described.

### 2.6. Analysis of the Enzymatic Activity of Allergen Native Extracts and Allergoids

The enzymatic activity of the NE was analysed by qualitative and quantitative methods. To analyse the qualitative enzymatic activity, nineteen enzymatic activities were analysed in the NE using ApiZym galleries (Biomérieux, Marcy-l’Étoile, France).

The proteolytic activity of the NE and PE was assessed by analysing the hydrolytic capacity of different types of enzymes: trypsin, serine protease, cysteine protease, and acidic and alkaline phosphatases. Specific substrates were used for each enzyme: Azocoll, S-2288 peptide, L-1460 peptide, and NPHP peptide. The absorbances of the coloured products obtained by the hydrolysis of these substrates were measured in a spectrophotometer and the values were extrapolated in a trypsin, papain, and acidic or alkaline phosphatase standard curve, as corresponding. The PEs were also analysed and compared to the native extract results.

To evaluate the effect of the proteolytic activity of the Alternaria extracts over grass allergen extracts, different mixtures of *Alternaria* and *Phleum pratense* (Inmunotek) were prepared and studied after incubation at different intervals of time.

### 2.7. Stability of Grass Extracts in Mixture with Alternaria Allergoids

Aqueous mixtures of *P. pratense* native extracts with native or polymerised *A. alternata* extracts were prepared. Three different types of extracts were prepared: (1) 500 μg/mL of the grass native extract dissolved in deionised water (used as controls); (2) 500 μg/mL of the grass native extract mixed with 25 μg/mL of the *Alternaria* NE; (3) 500 μg/mL of the grass native extract with 25 μg/mL of the mould PE. The grass extracts were kept at 4 °C, and their stability was evaluated, as described before, by protein quantification (Bradford method), protein profile (SDS-PAGE), Western blot, and ELISA competition, after 1 h, 1 week, 1 month, and 3 months of incubation. The immunodetection assays were developed using a commercial pool of sera (Plasmalab International, WA, USA) from patients containing specific IgE antibodies against the grass allergens, as corresponding.

## 3. Results

### 3.1. Protein and Allergenic Profile of the Extracts: SDS-PAGE and Immunoblotting

**Figure 1:**
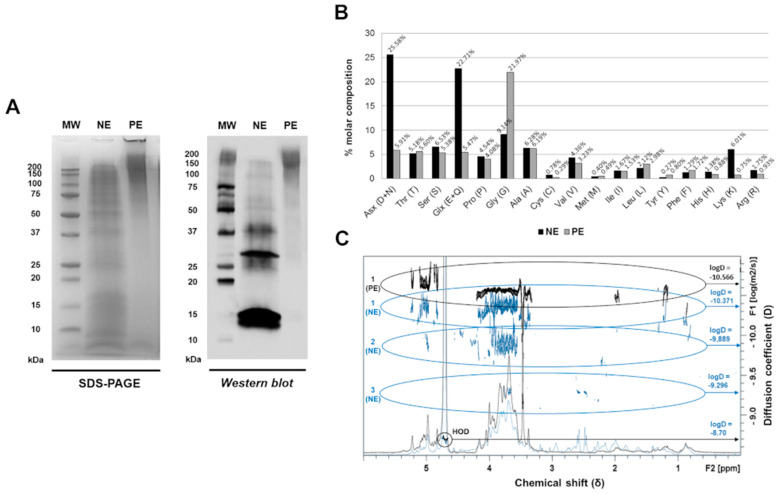
Protein and physicochemical characterisation of *A. alternata* native and polymerised extracts. (**A**) Protein (SDS-PAGE) and allergenic (Western blot) profiles of the native and polymerised extracts. Bands of varying molecular weights, corresponding to allergenic and non-allergenic proteins, were detected in the native extract (NE). In the polymerised extract (PE), the proteins appeared as a diffuse smear, predominantly >100 kDa. MW: molecular weight marker; NE: native extract; PE: polymerised extract. (**B**) The amino acid molar composition of native and polymerised extracts (%). The polymerised extract (PE) showed reduced detection of Lys, Arg, Asx, and Glx compared to the native extract (NE). The Gly content increased in the PE due to its use in stopping the polymerisation reaction. NE: native extract; PE: polymerised extract. (**C**) NMR analysis of *Alternaria alternata* native and polymerised extracts. Superimposed ^1^H NMR spectra and DOSY experiments are shown. The diffusion coefficient (D, m^2^/s) is displayed on the *y*-axis, with more negative values indicating larger hydrodynamic radii. The chemical shift (δ, ppm) is shown on the *x*-axis, highlighting carbohydrate regions (3.5–4.5 ppm) and protein regions, including aliphatic (0–3 ppm) and aromatic (4.5–5.5 ppm). NE: native extract (blue); PE: polymerised extract (black).

The protein content of the native extract (NE) displayed a broad range of protein sizes, with the molecular weights ranging from <10 kDa to >100 kDa, as revealed by the SDS-PAGE analysis (Figure 1A, left). At least six distinct protein bands showed IgE reactivity when probed with sera from allergic patients, as demonstrated by Western blotting (Figure 1A, right). Following polymerisation with 25% glutaraldehyde, the polymerised extract (PE) appeared as a diffuse smear in SDS-PAGE, with most proteins exceeding 100 kDa. Although some IgE reactivity was retained, virtually all the protein bands below 50 kDa, including the 16 kDa band likely corresponding to Alt a 1, were no longer detectable after polymerisation (Figure 1A).

### 3.2. Physicochemical Composition Analysis of the Extracts: Amino Acid Content, Nuclear Magnetic Resonance and Mass Spectrometry

Upon the addition of glutaraldehyde to the NE, the amine groups of proteins, primarily lysine (Lys), arginine (Arg), asparagine/aspartate (Asx), and glutamine/glutamate (Glx), underwent modification. These residues became undetectable in the PE by gas chromatography. Specifically, gas chromatography revealed reductions of 87.5% for Lys, 76.9% for Asx, 75.9% for Glx, and 46.9% for Arg in the PE, indicating successful polymerisation (Figure 1B).

The NMR-DOSY spectra of the NE revealed three distinct populations (1NE, 2NE, and 3NE), representing heterogeneous protein particles of varying molecular weights (Figure 1C). In contrast, the PE exhibited a single, homogeneous population (1PE) with a larger hydrodynamic radius than any NE population, indicating the formation of a uniform high molecular weight molecule. Despite structural changes induced by polymerisation, both the NE and PE exhibited similar carbohydrate (δ = 3.5–4.5 ppm) and protein (δ aliphatic = 0–2.5 ppm; δ aromatic = 4.5–5.5 ppm) composition profiles, as determined by NMR-DOSY. These findings suggest that glutaraldehyde polymerisation altered the protein structure without significantly affecting the overall chemical composition of the NE. The mass spectrometry analysis identified over 1000 proteins in the extracts. Seven allergens were detected in the NE, including Alt a 1, Alt a 3, Alt a 6, Alt a 10, Alt a 13, Alt a 14, and Alt a 15.

### 3.3. Allergenic Activity Analysis: IgE/IgG ELISA Competition Assays

To evaluate the allergenic activity of polymerised and native extracts, we performed ELISA competition assays.

**Figure 2:**
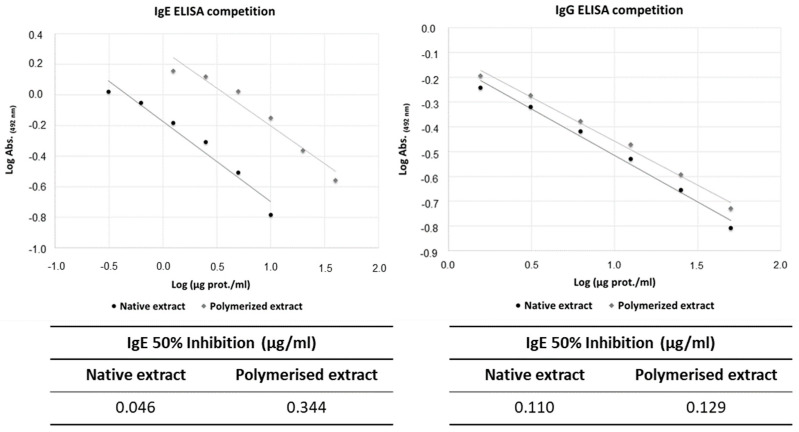
IgE- and IgG-binding capacities of the native and polymerised *A. alternata* extracts. Graphical representation of the IgE and IgG ELISA competition results for the native and polymerised extracts. The 50% inhibition values for each extract are summarised in the accompanying tables (µg/mL).

In the ELISA competition assays, NE inhibited e 50% of the IgE reactivity (IC50) to *A. alternata* at 0.046 µg NE/mL, while PE showed reduced activity, with 0.344 µg PE/mL (Figure 2, left panel). Regarding the IgG reactivity, the NE inhibited 50% at 0.11 µg NE/mL, while PE showed similar results, with 0.13 µg PE/mL to inhibit 50% of IgG reactivity to *A. alternata* (Figure 2, right panel). These results suggest that the polymerised extracts show an 87% reduction in allergenic activity while its immunogenicity seems to be conserved.

### 3.4. Immunogenicity and Inhibition of IgE Reactivity by Mouse IgG Antibodies Induced with A. alternata PE

**Figure 3:**
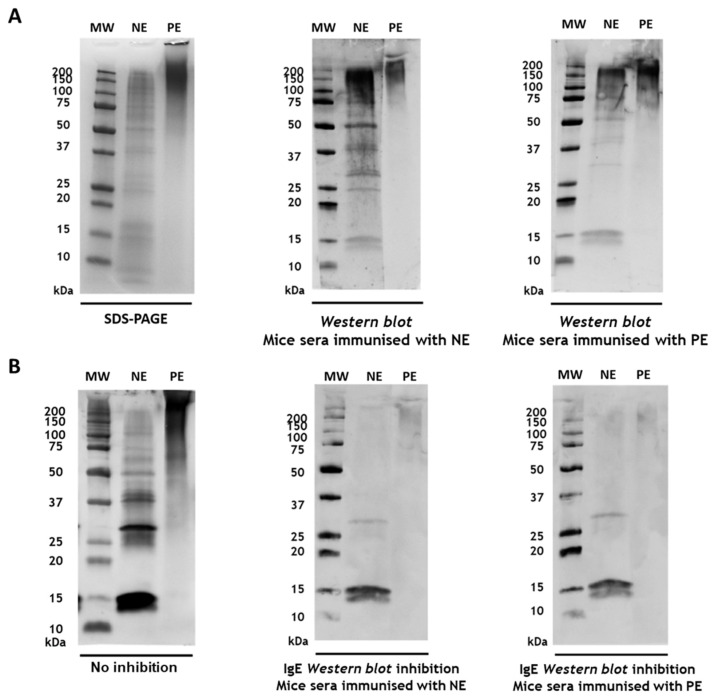
Inhibition of human IgE binding by mouse IgG induced with *A. alternata* allergoids (**A**) The Western blot analysis of IgG from mice immunised with the native (NE) or polymerised (PE) extracts, probed with NE and PE. (**B**) Western blot analysis of human IgE binding to NE and PE inhibited with sera from mice immunised with NE or PE. Immunisation with both the native and polymerised extracts induced antibodies capable of blocking the binding of IgE from allergic patients.

The Western blot analysis was performed to assess the immunogenicity of NE and PE of *A. alternata*, as well as the capacity of antibodies induced by these extracts to inhibit IgE binding from allergic patients. Mouse IgG antibodies induced by immunisation with *A. alternata* NE and PE were first probed against the respective extracts. In both cases, the antibodies strongly recognised multiple protein bands in the extracts, indicating robust immunogenicity for both NE and PE (Figure 3A).

To evaluate the ability of these IgG antibodies to inhibit IgE binding from human sera, the Western blot analysis of *A. alternata* NE and PE was conducted using human IgE pre-incubated with sera from immunised mice. IgE reactivity to native *A. alternata* allergens was significantly reduced in the presence of IgG antibodies generated through immunisation with PE (Figure 3B), comparable to the inhibition observed with IgG antibodies generated in the mice immunised with NE.

These findings demonstrate that the chemical modification of *A. alternata* extracts through polymerisation does not eliminate critical epitopes necessary for the induction of protective IgG antibodies.

### 3.5. Enzymatic Activity of Native and Polymerised Extracts

An enzymatic activity analysis using ApiZym kits detected 13 of 19 enzymatic activities in the *A. alternata* NE (Table 1). A quantitative analysis revealed that serine protease activity was the most abundant, followed by alkaline phosphatase, trypsin, and acid phosphatase activities, while cysteine protease activity was the least abundant. In the PE, all the enzymatic activities were significantly reduced compared to the NE. Trypsin and serine protease activities decreased by 99.73% and 90.05%, respectively, while acidic and alkaline phosphatase activities dropped by 71.33% and 75.5%. Cysteine protease activity was reduced by 59.15% (Table 2).

### 3.6. Stability of Grass Extracts in Mixture with Alternaria Allergoids

**Figure 4:**
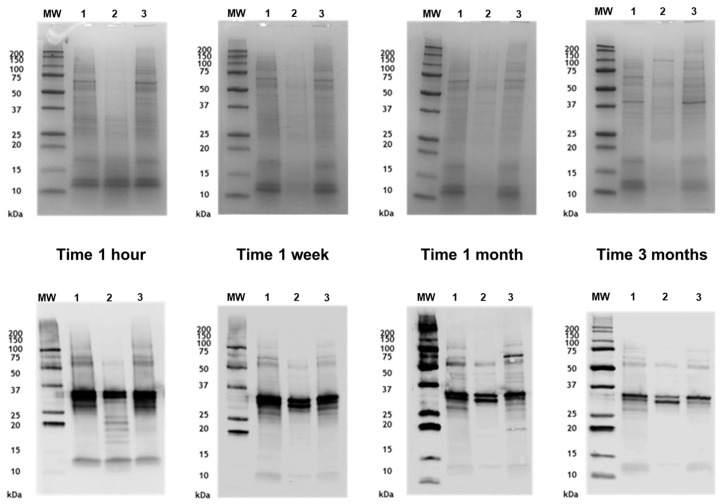
Stability of *P. pratense* protein and allergenic profiles in mixtures with native or polymerised *A. alternata* extracts. SDS-PAGE (**top**) and Western blot IgE (**botton**) of the *P. pratense* mixtures and control after different intervals of incubation. 1: Grass native extract; 2: *P. pratense* and *A. alternata* native extracts mixture; 3: *P. pratense* native extract and *A. alternata* polymerised extract mixture. MW: molecular weight marker.

The reduced proteolytic activity observed in the PE of *A. alternata* was confirmed in mixtures with grass (*Phleum pratense*) NE. The protein and allergenic profiles of the *P. pratense* mixtures were analysed at different incubation times and compared to the grass NE control dissolved in distilled water (Figure 4). In the mixtures of the two native extracts, the degradation of grass proteins was observed within 1 h, with the disappearance of bands >37 kDa compared to the control profile. After 3 months, this degradation became more pronounced, as indicated by reduced band intensity. In contrast, when the grass NE was mixed with the *A. alternata* PE, the protein profile remained stable and similar to the grass control throughout the study.

The allergenic profile of the grass control extract and the mixture with *A. alternata* PE showed a general decrease in band intensity at the 3-month time point.

**Figure 5:**
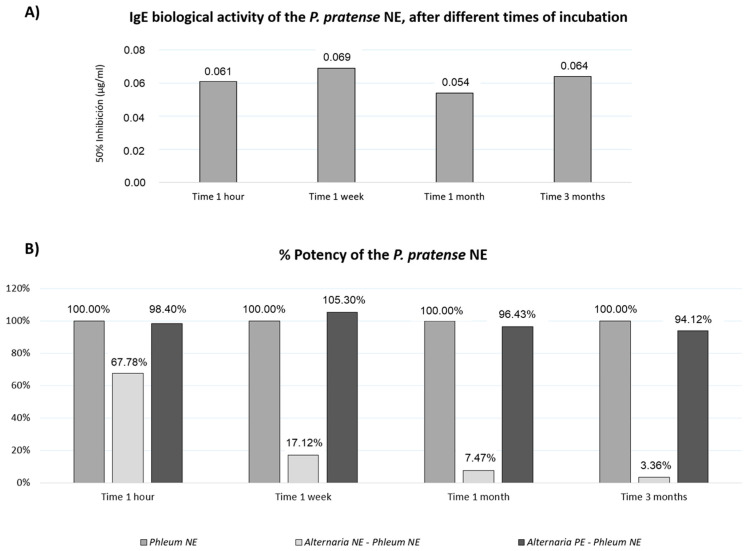
Preservation of *P. pratense* biological potency in mixtures with polymerised *A. alternata* extracts. (**A**) IgE biological activity (50% inhibition) of *P. pratense* native extracts (controls) over different incubation intervals. These values were set as the 100% potency reference for each interval to evaluate the mixtures’ potency. (**B**) Potency percentage of *P. pratense* native extract mixed with *A. alternata* native or polymerised extracts relative to the grass native extract (control) at different incubation intervals.

The biological IgE activity of the mixtures was evaluated by ELISA competition. The potency of the *P. pratense* control remained stable throughout the study (Figure 5A). However, when mixed with mould NE, grass potency dropped to 67.78% after 1 h and further declined to 3.36% after 3 months (Figure 5B). In contrast, the grass NE mixed with *A. alternata* PE maintained similar potency to the control throughout the study.

## 4. Discussion

A pivotal stage in allergen immunotherapy (AIT) involves the selection of the extract type, allergen content, and overall allergenic activity. The key factors contributing to treatment failures include inadequate allergen dosage, potential adverse effects, and the prolonged duration of treatment. The use of *A. alternata* extracts in AIT has been controversial due to challenges in standardisation and the associated high risk of adverse reactions [39,40]. In addition, *A. alternata* produces a broad and complex array of allergenic proteins [41] that collectively contribute to allergy symptoms [42], potentially limiting the effectiveness of single-allergen immunotherapy in patients sensitised to multiple *A. alternata* allergens. Native fungal allergenic extracts have a complex composition of proteins, carbohydrates and other components that do not contribute to allergenicity but may produce adverse effects during treatment, so the safety of subcutaneous immunotherapy with fungi native extracts was questioned [42]. To mitigate secondary adverse reactions, strategies such as hypoallergens, peptide-derived vaccines, or purified allergens have been developed. Nevertheless, there is a need for further studies confirming the effectiveness of immunotherapy with *Alternaria* extracts, particularly in relation to long-term efficacy and safety [43,44].

In this study, we demonstrate that glutaraldehyde-treated *A. alternata* extracts can generate allergoids with reduced IgE-binding capacity while preserving immunogenicity across various allergens, including Alt a 1. These allergoids represent a safer and more effective alternative to conventional vaccines for *A. alternata* allergy treatment.

The mechanisms underlying the advantages of allergoids over native extracts in AIT are not fully elucidated. Current evidence suggests that chemical modification reduces IgE-binding capacity while retaining recognition by IgG and linear T-cell epitopes, facilitating a protective immune response characterised by strong IgG production and/or regulatory IL-10-dependent T-cell responses [45,46,47,48]. However, it is increasingly evident that specific IgG production alone does not guarantee protection. Factors such as epitope specificity and IgG affinity play crucial roles in determining therapeutic efficacy [49,50,51]. Maintaining the native epitope repertoire during allergenic extract modification is critical for generating blocking IgG antibodies that specifically inhibit IgE-allergen interactions.

We demonstrate that mouse IgG antibodies, generated through immunisation with *A. alternata* allergoid, exhibit the capacity to inhibit the reactivity of native allergens present in the extract against human IgE from allergic patients. These results are important because indirectly indicate that the chemical modification did not affect the epitopes or prevent them from inducing a protective immunological response based on the induction of blocking IgG.

The use of a complete source of *Alternaria* antigens offers an advantage over single-allergen immunotherapy. As aforementioned, Alt a 1 is the most important *Alternaria allergen*, but it is not the unique sensitising allergen. There are studies that show the great relevance of some of the other minor allergens, such as Alt a 13, Alt a 4, and Alt a 8 [52]. Our immunoblotting results indicate that the other *Alternaria* proteins are recognised by IgE from sensitised patients. The prevalence of the sensitisation of other *Alternaria* allergens and their relevance in symptomatology has not been well studied. As an exception, Armentia et al. found that 46.7% of patients with an allergy to fungi were sensitised to Alt a 6 following a molecular analysis [53]. Recently, Rodriguez et al. observed that among 64 patients, the percentage of recognition for Alt a 3, Alt a 4, and/or Alt a 6, Alt a 7, Alt a 8, Alt a 10, and/or Alt a 15 was 1.6%, 21.9%, 12.5%, 12.5%, and 12.5%, respectively. Notably, 30% of the patients exhibited recognition of several *Alternaria* allergens beyond Alt a 1 [54]. Furthermore, sensitisation to these allergens may play a crucial role in pathology. Illustratively, in a study involving 100 atopic dermatitis patients, sensitisation to Alt a 6 was found to be associated with a subgroup of individuals suffering bronchial asthma [55].

Fungal enzymes play an important role in allergy, both indirectly, by facilitating the passage of other allergens across the epithelial barrier, and directly, by acting on dendritic cells and promoting Th2-type responses. The cross-linking of enzymes with GA leads to a series of structural changes in the enzymes that result in decreased proteolytic capacity and possibly their allergenicity. Enzyme analysis of the extracts showed a significant decrease (*p* < 0.001) in proteolytic activity in the polymerised extract compared to the native extract. The most significant decrease was observed in serine protease activity (including trypsins), with more than 90% loss of the proteolytic capacity. Phosphatase activity also showed a 71–75% reduction, while cysteine protease activity (59.15%), which are less abundant in the extract.

The reduced enzymatic activity of *A. alternata* allergoids has important implications for polysensitised patients. Fungal enzymes in native extracts are known to degrade allergens from other sources when combined in aqueous mixtures [56,57,58,59]. Our findings demonstrate that *P. pratense* allergens were degraded within an hour when mixed with native *A. alternata* extracts, resulting in a 33.2% reduction in biological potency after the first hour of incubation. In contrast, mixtures with polymerised *A. alternata* extracts maintained the stability and potency of grass allergens, as confirmed by electrophoresis and ELISA competition assays. Kordash et al. obtained similar results for extracts of Lolium perenne in a glycerol mixture with extracts of the fungus *Helmintosporium* spp. by observing a decrease in potency, as assessed by RAST. However, the biological activity estimated by skin tests was not altered [60].

## 5. Conclusions

Polymerising *A. alternaria* native extracts results in hypoallergenic allergoids that induce robust IgG responses that can potentially regulate the allergic response generated after natural exposure to *A. alternaria*. These polymerised allergens constitute an alternative to replace native allergenic extracts for AIT and can be considered to be co-administered in a mixture with grass allergy treatments.

## Figures and Tables

**Table 1:** Qualitative determination of the enzymatic activity in *A. alternata* native extracts (+: detected, -: not detected).

Enzymatic Activity	Native Extract
Alkaline phosphatase	+
Esterase (C4)	+
Esterase lipase (C8)	+
Lipase (C14)	-
Leucine arylamidase	+
Valine arylamidase	-
Cystine arylamidase	-
Trypsin	+
α-chymotrypsin	-
Acid phosphatase	+
Naphthol AS-BI-phosphohydrolase	+
α-galactosidase	+
β -galactosidase	+
β-glucuronidase	-
α-glucosidase	+
β-glucosidase	+
N-acetyl-β-glucosaminidase	+
β-mannosidase	+
α-fucosidase	-

**Table 2:** Results of the quantification of the specific proteolytic activities assessed in the *A. alternata* native and polymerised extracts (expressed in enzyme equivalents/protein mg). The percentage of diminution refers to the diminution of the proteolytic activity in the polymerised extract compared to the native extract.

Proteolytic Activity	Native Extract	Polymerised Extract	% Diminution
Trypsin	119.049	0.321	99.73
Serine protease	6172.52	614.03	90.05
Cysteine protease	3.55	1.45	59.15
Acid phosphatase	98.62	28.27	71.33
Alkaline phosphatase	543.86	134.80	75.21

## Data Availability

This study did not generate a publicly archived dataset.
